# Pathogenesis, diagnosis and therapy of facial synkinesis: A systematic review and clinical practice recommendations by the international head and neck scientific group

**DOI:** 10.3389/fneur.2022.1019554

**Published:** 2022-11-09

**Authors:** Orlando Guntinas-Lichius, Jonas Prengel, Oded Cohen, Antti A. Mäkitie, Vincent Vander Poorten, Ohad Ronen, Ashok Shaha, Alfio Ferlito

**Affiliations:** ^1^Department of Otorhinolaryngology, Jena University Hospital, Jena, Germany; ^2^Facial Nerve Center, Jena University Hospital, Jena, Germany; ^3^Multidisciplinary Salivary Gland Society, Geneva, Switzerland; ^4^Department of Otolaryngology, Head and Neck Surgery, Soroka Medical Center, Affiliated With Ben-Gurion University of the Negev, Be'er Sheva, Israel; ^5^Department of Otorhinolaryngology-Head and Neck Surgery, Helsinki University Hospital, University of Helsinki, Helsinki, Finland; ^6^Department of Oncology, Section Head and Neck Oncology, KU Leuven, Leuven, Belgium; ^7^Otorhinolaryngology, Head and Neck Surgery, Leuven Cancer Institute, University Hospitals Leuven, Leuven, Belgium; ^8^Department of Otolaryngology-Head and Neck Surgery, Galilee Medical Center, Affiliated With Azrieli Faculty of Medicine, Bar-Ilan University, Safed, Israel; ^9^Head and Neck Service, Memorial Sloan Kettering Cancer Center, New York, NY, United States; ^10^International Head and Neck Scientific Group, Padua, Italy

**Keywords:** Bell's palsy, electromyography, aberrant regeneration, botulinum toxin, surgery, EMG feedback training, electrostimulation, facial synkinesis

## Abstract

**Introduction:**

Post-paralytic facial synkinesis after facial nerve injury produces functional disabilities and mimetic deficits, but also cosmetic and non-motor psychosocial impairments for the patients. These patients typically have a high and continuous high motivation for rehabilitation. The aim is to inform the affected patients and their therapeutic professionals (otorhinolaryngologist - head and neck surgeons; oral-maxillofacial surgeons, plastic and reconstructive surgeons, neurosurgeons, neurologists, and mime therapists be it speech and language therapy- or physiotherapy-based) and to provide practical recommendations for diagnostics and a stepwise systematic treatment approach of facial synkinesis.

**Methods:**

In the first phase, a systematic literature search on the topic in PubMed and ScienceDirect starting in 2008 resulted in 132 articles. These were the basis for the review and a comprehensive series of consensus statements on the most important diagnostic tests and treatment options. In the second phase, one consensus article circulated among the membership of the International Head and Neck Scientific Group until a final agreement was reached for all recommendations.

**Results:**

Diagnostics should include a standardized assessment of the degree of synkinesis using validated clinician-graded instruments and synkinesis-specific patient-reported outcome measures. Treatments for facial synkinesis include facial training mainly based on facial biofeedback retraining, chemodenervation with botulinum toxin, selective neurectomy, myectomy, and any combination treatment of these options.

**Conclusion:**

A basic understanding of the pathomechanisms of synkinesis is essential to understand the treatment strategies. A standardized assessment of the synkinetic symptoms and the individual synkinesis pattern is needed. The first-line treatment is facial training, followed by botulinum toxin. Surgery is reserved for individual cases with unsatisfactory first-line treatment.

## Introduction

Facial synkinesis is a condition characterized by unintentional facial muscle activation and the resulting mimic movement occurring simultaneously with intentional facial movements (1). Patients are often unable to fully control perioral and midfacial muscles resulting in problems during eating, drinking, and facial expression (1). Involuntary eye closure during intentional mouth movements while speaking or eating is considered a significant disturbing outcome. This leads to both aesthetic and functional deficits, placing patients at risk for psychosocial sequelae and social impairment (2). The basis for the optimal personalized treatment of facial synkinesis is a fundamental understanding of the underlying pathophysiological mechanisms. Since the first comprehensive review by Crumley in 1979, our current knowledge of the background, especially on the role of cortical control in facial synkinesis, has increased (3). A recently published book on new diagnostics and therapy techniques makes detailed contributions to the current knowledge of facial synkinesis (4). The present guideline by international experts in the field gives practical recommendations for the treatment of patients with facial synkinesis based on a systematic review of the literature and a consensus process. Despite the increasing research in the field, guidelines for the management of synkinesis have yet to be published. The aim of this systematic review is to establish the base for a possible guideline, which would serve relevant providers (i.e., otorhinolaryngologists - head and neck surgeons and other medical specialists, therapeutic professionals, and the affected patients), as well as to provide practical recommendations for diagnostics and a graduated therapy of facial synkinesis. Because understanding the pathophysiology of the development of facial synkinesis is important to realize the diagnostic needs and treatment concepts, the current state of knowledge on the pathophysiology was placed in front. Thereafter recommendations on diagnostics and therapy are presented. Specific clinical grading systems of facial synkinesis, sophisticated electrophysiological testing, and patient self-reported outcome measures allow for a precise definition of the patient's individual physical and psychosocial burden. The ultimate aim is to provide an individually staged treatment concept consisting of non-surgical and surgical measures.

## Materials and methods

### Search and consensus strategy

**Figure 1:**
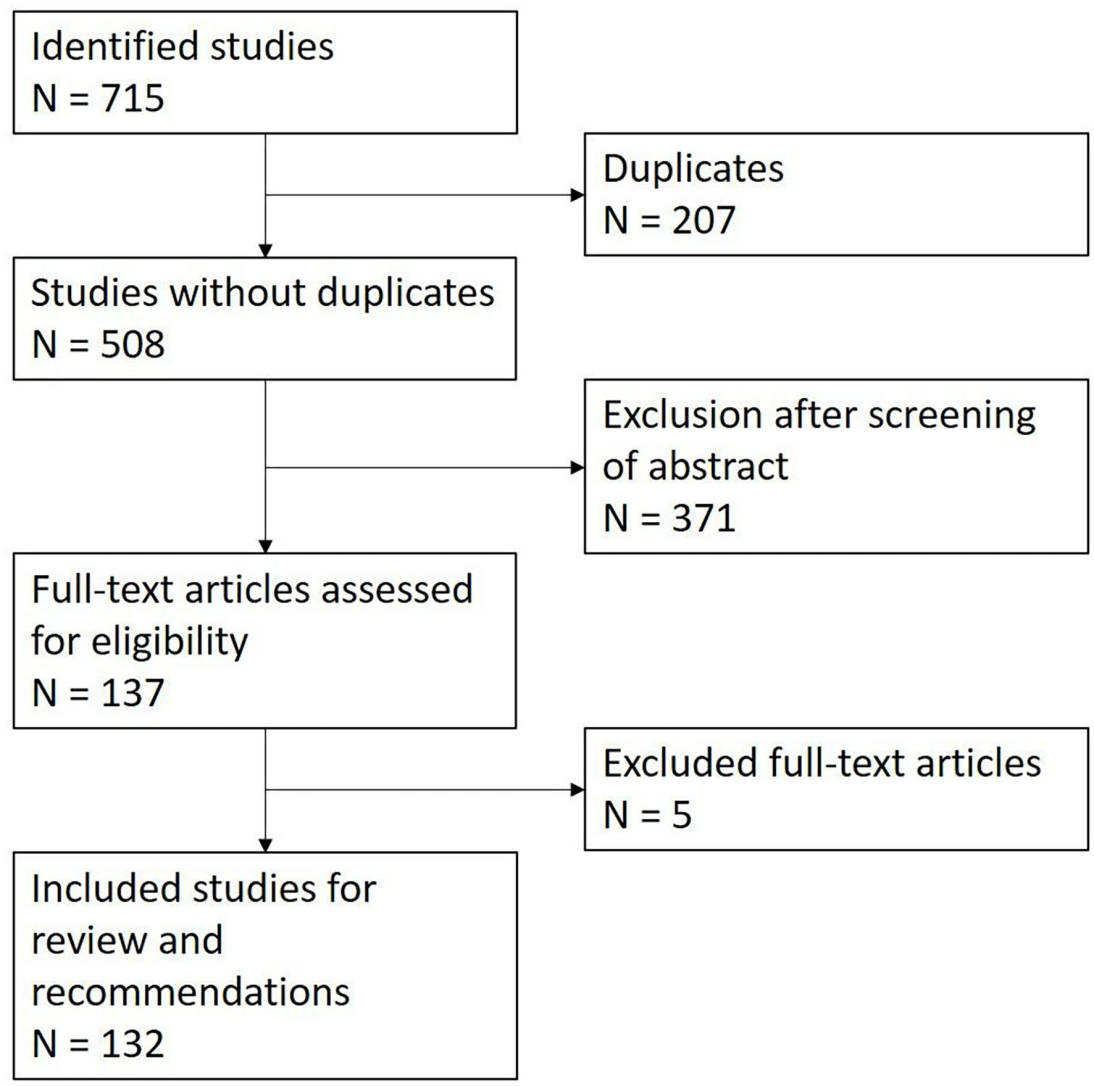
Flow diagram of the literature selection process.

As a starting point, a careful review of a highly cited narrative review on facial synkinesis published in 2008 was conducted (5). This was followed by a systematic review conducted in three steps in accordance with the preferred reporting items for systematic reviews and meta-analyses (PRISMA) guidelines (6). We conducted a systematic literature search for publications in the English language since 2008 using PubMed and ScienceDirect database, with the following MeSH terms: “facial nerve,” “Bell's palsy,” “facial nerve diseases,” “synkinesis,” “post-paralysis,” “post-paralysis,” “facial palsy,” “facial paralysis,” “facial nerve injuries,” “post-paralytic facial nerve syndrome,” “synkinetic smile,” “aberrant regeneration,” and “humans” (period: 2008–2022; last search on 05-05-2022). In addition, the recent search results from Lapidus et al. used in their systematic review of facial synkinesis treatment were reviewed (7). Lapidus et al. retrieved 1250 records up to February 2018 and the Delphi consensus study on outcome measures for facial synkinesis by Berner et al. revealed 502 studies with large overlap to the retrieval of Lapidus et al. (7) and Berner et al. (8). Our research from 2018 to May 2022 retrieved another 208 records. In agreement with the PRISMA guidelines (6), we reported the results using the PICOST-DS tool (9): Participants: all ages, affected with facial synkinesis; intervention: any kind of intervention: diagnostics and therapy; comparator: not needed; outcomes: no restriction; time: no limits of the time; setting: outpatients and inpatients; and study design: all designs studies. Finally, a total of 132 manuscripts were included in the present review based on relevance and scientific evidence. A flow diagram of the research is reported in Figure 1.

### Recommendation assessment

The highest level of evidence reached the level of retrospective observational cohort studies (Oxford Centre for Evidence-based Medicine Level III-IV). Due to the lack of higher quality evidence, the presented recommendations reached the level of recommendation B, i.e., considerable benefit substantiated by non–first-class evidence, according to international standards and the Association of the Scientific Medical Societies guidelines (Arbeitsgemeinschaft Wissenschaftlich Medizinischer Fachgesellschaften, AWMF; https://www.awmf.org/en/clinical-practice-guidelines/awmf-guidance.html). The most important diagnostic tests and treatment options were discussed in-depth and a consensus was proposed. The manuscript circulated among the authors in two rounds until a consensus was reached for all recommendations. A strong consensus (agreement among > 95% of participants) was reached for all recommendations based on the Delphi process. A relevant part of the available literature regards exclusively the sequelae of patients with idiopathic facial palsy (Bell's palsy). If a recommendation applies solely to patients who suffered from idiopathic facial palsy, this is highlighted in the text.

## Results

### Definition of facial synkinesis

Facial synkinesis (post-paralytic facial synkinesis; post-paralytic facial nerve syndrome with synkinesis) is a symptom in which a voluntary facial muscle movement causes the simultaneous involuntary contraction of other facial muscles, which can occur in all facial muscles (10). Because of the functional importance, synkinesis between ocular and oral facial muscles receives more attention and is the focus of treatment. This should be referred to as oculo-oral synkinesis.

### Pathophysiology of facial synkinesis

A pre-condition for the development of facial synkinesis is the damage of facial nerve axons (as opposed to neuropraxia) and regrowth of these axons, which can be detected by electrophysiological tests in the acute stage (11, 12). Vice versa, this means that synkinesis cannot occur without axonal damage. Many patients with Bell's palsy fortunately only develop neurapraxia, i.e., a transient blockage of facial nerve conduction without axonal damage (13). These patients cannot develop synkinesis. Only those patients with Bell's palsy and axonal damage can develop facial synkinesis. The synkinesis is a result of misguided nerve regeneration following axonal damage. This is the most widely accepted mechanism. Nevertheless, it has been proposed that hyperexcitability of the facial nucleus, ephaptic nerve transmission, and higher cortical re-organization may also contribute to facial synkinesis (4). Table 1 summarizes the pathomechanisms of facial synkinesis. The main cause of facial nerve damage is a nerve lesion due to nerve trauma. Facial synkinesis cannot occur without the regeneration of the damaged axons and reinnervation of facial muscles. This can occur spontaneously or be induced by facial nerve repair.

**Table 1:** Proposed elements of the pathomechanism of facial synkinesis.

**Mechanism**	**Comment**
Collateral sprouting leading to misguided peripheral nerve regeneration	Most damaged axons will develop several sprouts and will regrow randomly into different peripheral facial nerve branches. Facial synkinesis occurs when one axon sends sprouts to muscle fibers of two different muscles, i.e., the original target muscle and any other muscle. This phenomenon is proven in humans by electrophysiological studies.
Terminal sprouting as part of the pathological peripheral nerve regeneration	Polyneuronal innervation by axonal sprouting in the motor end plate region is another sprouting phenomenon seen during facial nerve regeneration. This has been shown in animal studies. The effect in patients is unclear.
Post-damage hyperexcitability of the facial nucleus	Synaptic stripping occurs very early after facial nerve injury but seems to be not completely reversible after regeneration. This leads to a permanent imbalance between excitatory and inhibitory input on the facial motor neurons. This has been shown in animal studies and in human post-mortem studies.
Ephaptic nerve transmission after facial nerve regeneration	If the re-myelination after axon regeneration following an injury is incomplete, an axon can theoretically cross-excite an adjacent axon *via* ephaptic coupling. This phenomenon is known from patients with hemifacial spasms, but has not clearly been proven for patients with facial synkinesis.
Higher cortical re-organization after facial nerve lesion and regeneration	There is a permanent functionally disturbed cortical motor control in patients with facial synkinesis as revealed by human magnetic resonance imaging (MRI) and functional MRI (fMRI) studies.

#### Misguided nerve regeneration

The hypothesis of misguided nerve regeneration assumes that disorganized repair of the damaged facial axons occurs at the site of the lesion [Details in: (4, 14)]. Some axons fail to regrow. Some axons may only regrow with one sprout and will reach the muscle fiber of one target muscle as the original. Most axons will develop several sprouts (also called collateral sprouting) and will regrow randomly into different peripheral facial nerve branches. Facial synkinesis occurs when one axon sends sprouts to muscle fibers of two different muscles, i.e., the original target muscle and any other muscle. There is one animal study that has also shown that this misguidance can occur throughout the complete peripheral length of the facial nerve (15). This has not been confirmed by others or in patients. Terminal sprouting, i.e., polyneuronal innervation by axonal sprouting in the motor end plate region is another sprouting phenomenon seen during facial nerve regeneration and muscle reinnervation in animal studies (16). In humans, terminal sprouting has only been confirmed for synaptic displacement after botulinum toxin injections into facial muscles and does promote facial synkinesis (17). If a regenerating axon is randomly sending several collateral sprouts into the same facial muscle, hyperkinesis can occur where the muscle activity is too strong. This phenomenon is also being held responsible for hypertonia in facial muscles seen in some patients. If the synkinetic activation affects antagonistic muscles, the movement can be neutralized and can create the false appearance of muscle paralysis (autoparalytic syndrome) (18). The best example is the persistent loss of frowning on the lesioned side. The reason in most cases is an antagonistic co-activation of the frontalis and orbicularis oculi muscle. Sometimes, patients also develop involuntary twitching of facial muscles, usually without noticing themselves. Those are characterized by spontaneous unintentional very tiny muscle fiber fibrillations without the contraction of the entire affected facial muscle. It is assumed that this twitching is triggered by misdirected facial motor neurons originally responsible for eye blinking.

#### Ephaptic transmission and increased excitability of the facial nucleus

If the re-myelination after axon regeneration following an injury is incomplete, an axon can cross-excite an adjacent axon *via* ephaptic coupling. A co-excitation of different muscles would theoretically also lead to synkinesis. Although this mechanism is listed in nearly every review of facial synkinesis, there is no clear proof (19). In contrast, synaptic stripping in the facial nucleus as the cause of hyperexcitability in the facial nucleus has been proven post-mortem in a patient with facial synkinesis (20). Synaptic stripping occurs very early after facial nerve injury but seems to be not completely reversible after regeneration. This leads to a permanent imbalance between excitatory and inhibitory input on the facial motor neurons (21).

#### Cortical re-organization

Several human magnetic resonance imaging (MRI) and functional MRI (fMRI) studies in recent years have supported the role of functionally disturbed cortical motor control in patients with facial synkinesis (22, 23). This may help to develop new treatment concepts. Even transient peripheral de-efferentation in the case of Bell's palsy with complete clinical recovery leads to altered functional connectivity in the cortex contralateral to the formerly paretic side (24). Compared with healthy controls, patients with facial synkinesis demonstrated a decreased activation in the cortico-facial motor representation area, while being increased in the supplement motor area during tasks (25). In addition, patients with unilateral facial synkinesis show decreased distances between the cortical representation sites during blinking and smiling tasks (26). This is accompanied by a pathological coupling of cortical areas during facial tasks (23). The local synchronization in motion-related brain regions is decreased (22). There seems to also be an irreversible structural remodeling of gray matter in patients with facial synkinesis. The contralateral superior and inferior temporal gyri show a reduced cortical thickness (27).

*Recommendation:* Facial synkinesis is the result of a complex pathological regeneration with changes in the brainstem nucleus and the higher cortex. Facial synkinesis can occur only after pathological regeneration, distinguishing it from its pre-condition, facial palsy, or paralysis. Facial synkinesis (also called post-paralysis or post-paralytic facial synkinesis) is considered a muscle movement coordination disorder and should be addressed as such.

### Clinical assessment and grading of facial synkinesis

**Figure 2:**
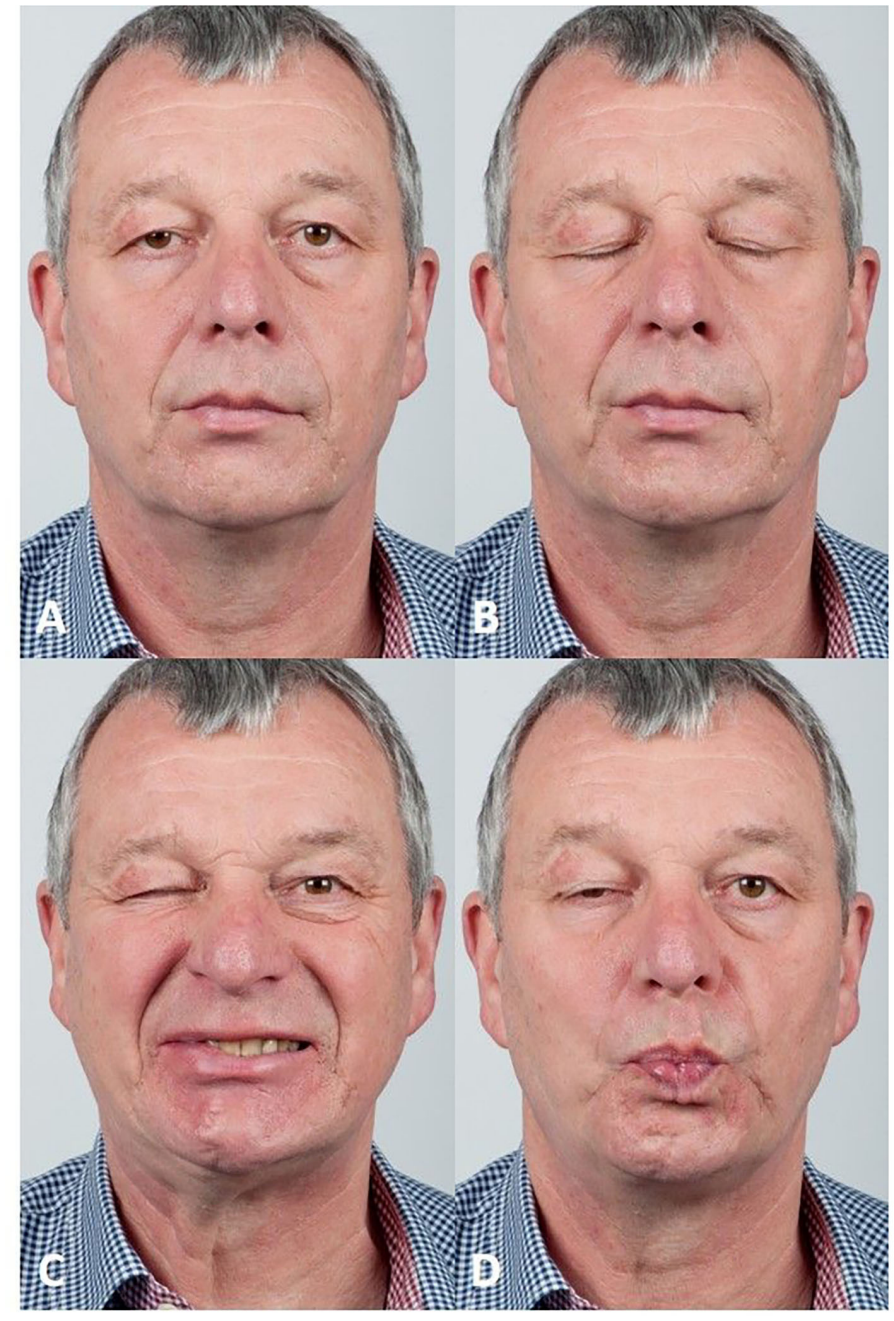
Example of typical signs of synkinesis at rest and during movements. **(A)** At rest, a pronunciation of the nasolabial fold on the post-paretic right side and slight ptosis of the upper eyelid is visible. **(B)** With the exception of a synkinetic activity in the region of the right corner of the mouth, especially in the zygomatic region, eye closure is not accompanied by strong synkinetic muscle activity. Furthermore, disturbing synkinesis of the right depressor anguli oris of the platysma can be observed. **(C)** The severe oculo-oral synkinesis becomes obvious during smiling leading to a nearly complete involuntary closure of the right eye. **(D)** The same can be seen when the patient is pursing the lips.

Table 2 lists the most important assessment tools and outcome measures. The abnormal and unintentional co-contractions of facial muscles are the characteristics of facial synkinesis. The most common complaint of facial synkinesis is unintentional eye narrowing or closure during voluntary mouth movements (e.g., smile, kiss, or blowing) or, vice versa, unintentional midface muscle contraction during voluntary eye closure (oculo-oral synkinesis; Figure 2). Synkinesis can be present in all facial muscles including the platysma, leading to uncomfortable neck contraction (35), and even the external and internal ear muscles (36). When intra-temporal or a more proximal lesion occurs, stapedial synkinesis can occur, leading to changes in the hearing threshold (37). Another rare symptom is gustatory hyperlacrimation (crocodile tears) induced by misdirected reinnervation of gustatory fibers through the greater superficial petrosal nerve to reach the lacrimal gland. This results in tearing when the patient eats (38). This can be proven by a modified Schirmer test (Schirmer test while eating).

**Table 2:** Recommended assessment tools and outcome measures.

**Tool**	**Comment**
Clinical assessment	Examination at rest for hyperkinesis and muscle twitching and with standard sequence of facial exercises
Photo and video documentation (28)	Documentation of the assessment for follow-up and facial grading
**Facial synkinesis grading**	
Sunnybrook (29)	Evaluation of the resting symmetry, degree of voluntary movement, and synkinesis to form a composite score
eFACE/auto-eFACE (30, 31)	Evaluation of three categories with static, dynamic, and synkinesis parameters. The auto-eFACE is a machine learning–derived automated assessment tool of the original eFACE.
**PROMs**	
SAQ (32)	The only available specific PROM for the evaluation of synkinesis.
FaCE (33).	Popular facial nerve specific PROM.
FDI (34).	Popular facial nerve specific PROM.
Electromyography (12)	Multichannel electromyography recording synchronously from several facial muscles during facial exercises prove the presence of facial synkinesis.
Modified Schirmer test	In case of gustatory hyperlacrimation (crocodile tears).
Hearing tests	In case of synkinesis of the stapedius muscle.

PROM, patient-reported outcome measure; SAQ, Synkinesis Assessment Questionnaire (SAQ); FaCE, Facial Clinimetric Evaluation; FDI, Facial Disability Index.

Facial synkinesis does not only become obvious during facial expressions, but also at rest, the patients can show intermittent or reduced blinking on the affected side or intermittent cheek muscle twitching. Hyperkinesis may look like muscle contracture with a deep nasolabial fold or permanent lower lip retraction. Hyperkinesis is often felt as a sensation of a tight face. If patients show areas with movement weakness, it has to be proven that the affected agonist and antagonist facial muscles do not result in pseudo-paralysis. Only a few popular grading systems allow for a classification of the degree of synkinesis. For instance, the still very popular House–Brackmann grading system only includes a gross description of the severity of synkinesis (no synkinesis, no disfiguring synkinesis, or synkinesis) (39). The updated House–Brackmann system, the Facial Nerve Grading System 2.0, introduced the category “secondary movement” for four regions: brow, eye, nasolabial fold, and oral with a 4-point Likert scale (0 = no synkinesis; 3 = disfiguring synkinesis) (40). The Sunnybrook system evaluates resting symmetry, degree of voluntary movement, and synkinesis to form a composite score (29). The synkinesis score grades synkinesis from 1 (mildest) to 15 (most severe) and allows a regional synkinesis grading (during brow lift, gentle eye closure, open mouth smile, snarl, lip pucker). The eFACE has three categories with static, dynamic, and synkinesis parameters (30). It also contains a regional synkinesis assessment (ocular, midface, mentalis, platysmal) on a visual analog scale from 0 (severe) to 100 (absent). Further development of the eFACE is a machine learning-derived automated assessment tool (auto-eFACE) for automated eFACE synkinesis scoring (31).

The two popular facial nerve-specific patient-reported outcome measures (PROMs), the Facial Clinimetric Evaluation (FaCE) and the Facial Disability Index (FDI) do not address synkinesis (33, 34). The Synkinesis Assessment Questionnaire (SAQ) is a specific PROM for the evaluation of synkinesis (32). The SAQ has nine questions addressing the regions of synkinesis. The final synkinesis grading should not be performed 12–15 months after the onset of the lesion as it takes this time to reach the final synkinesis state (41). The clinician might have a different view on the functionally relevant synkinetic facial areas than the patient. The SAQ displays the patients' perceptions of the important synkinetic symptoms (42).

The use of PROMs such as the FaCE, FDI, and SAQ will show the clinician that many patients with facial synkinesis also feel a severe reduction in quality of life (43–45).

*Recommendation*: A standardized assessment of the degree of synkinesis allows for a better comparison of patients and an optimal treatment evaluation. Validated clinician-graded instruments are the Sunnybrook facial grading scale and the eFACE. Use one of these tools to classify the patient's synkinesis. The auto-eFACE tool even allows automated observer-independent synkinesis scoring. The Synkinesis Assessment Questionnaire (SAQ) is the only available synkinesis-specific PROM and should be used to assess the patient's view on their synkinesis severity.

### Electrophysiological diagnostic work-up of facial synkinesis

Depending on the lesion site and severity of the facial nerve damage, electromyography (EMG) of the facial muscles will first show polyphasic reinnervation potentials 4–10 months after the onset of the lesion. With increased reinnervation and a time delay of about 2 months after the onset of the polyphasic reinnervation potentials, EMG will show facial synkinesis as the result of collateral sprouting (12). During EMG (mostly as needle EMG, but surface EMG can also be feasible), the synkinetic activity can be shown by placing the needle electrode in one muscle and having the patient move another facial muscle. Alternatively, a 2-channel or multiple-channel recording is used. Two or more needle electrodes are placed in different muscles. A firing of one motor unit action potential in two or more different muscles is recorded during voluntary contraction and proves synkinetic activity. By doing so, all relevant muscle interactions can be quantified. When the reinnervation process is finished, i.e., about 12–15 months after a lesion in the region of the facial main trunk, synkinesis has reached its final state (46).

*Recommendation*: Electromyography allows for a detailed analysis of the synkinetic pattern on the level of each facial muscle. It should be a standard diagnostic tool in patients with facial synkinesis who wish a therapy. EMG can detect synkinetic activity much earlier and more precisely than clinical examination alone. It can also be useful during the early stages of reinnervation.

### Prevention of facial synkinesis

About 70–90% of all idiopathic facial palsy cases recover in 12 months when treated under the standard corticosteroid therapy, i.e., about 10–30% develop synkinesis (41). The synkinesis rate is higher (40%) among patients with proven axonal injury (47). About 6.6% of idiopathic facial palsy cases develop moderate-to-severe synkinesis, defined by a Sunnybrook synkinesis score of ≥ 6 (41). Beyond corticosteroid therapy, there is no other well-established treatment for the acute phase after facial nerve lesions to prevent facial synkinesis (48). The role of antiviral treatment still is unclear. There is no high evidence that the addition of antivirals to corticosteroids can further decrease the synkinesis rate (49, 50). Furthermore, there is no evidence for corticosteroid therapy for other etiologies than idiopathic facial palsy. That is, there is no evidence of corticosteroid therapy for post-surgical facial nerve lesions with spontaneous regeneration or induced by nerve repair. Two small studies with several methodological limitations failed to show that the synkinesis rate after biofeedback electromyography therapy in the phase of acute palsy was significantly better than common physiotherapy (51, 52). In a small randomized trial on unselected cases with mild-to-severe Bell's palsy, the synkinesis rate after prednisolone and/or acyclovir plus electrical stimulation was lower (4%) than after drug treatment only (12%) (53). Puls et al. compared patients with spontaneous regeneration without and with surface electrical stimulation after severe degenerative facial nerve lesions (54). Synkinesis was significantly reduced when using electrical stimulation. More data are needed to confirm that electrical stimulation can reduce the occurrence of facial synkinesis. Table 3 lists all the important measures to prevent facial synkinesis.

**Table 3:** Most important measures to prevent facial synkinesis based on this review.

**Measure**	**Comment**
Corticosteroid therapy	First-line evidence-based treatment of acute idiopathic facial palsy. After treatment, 10–30% of patients develop facial synkinesis.
EMG-biofeedback exercises	Primarily developed for patients with facial synkinesis (see Table 2). Preliminary small studies also show an effect to reduce the probability of facial synkinesis when applied in the acute phase of facial palsy. More studies are needed to confirm such an effect before it can be recommended for clinical routine use.
Facial electrostimulation	Preliminary small studies show an effect to reduce the probability of facial synkinesis when applied in the acute phase of facial palsy. More studies are needed to confirm such an effect before it can be recommended for clinical routine use.
Facial nerve reconstruction with a combined approach	In case of extratemporal facial nerve reconstruction, one option to reduce synkinesis development is to divide the reconstruction of the upper and lower face to different motor nerves, i.e., to use the ipsilateral proximal facial nerve, the masseteric nerve, the hypoglossal nerve as jump, the ansi hypoglossi, and/or branches of the contralateral facial nerve together for facial reinnervation.
Facial nerve reconstruction with selective neurectomy	Selective neurectomy was originally established as second/third line treatment of facial synkinesis (see Table 5). This can also be performed together with the facial nerve reconstruction to prevent the sprouting of the regenerating axons into selected peripheral nerve branches. This can only be recommended for surgeons with high experience in facial nerve reconstruction.

*Recommendation:* Initiation of corticosteroid therapy within 72 h after the onset of idiopathic facial palsy with more than mild dysfunction (House–Brackmann grade >I or Sunnybrook score <90) is recommended to lower the risk of development of synkinesis. The evidence for corticosteroid therapy for non-idiopathic facial palsy is low.

The ability of other combinations of corticosteroids with electrical stimulation or sequential therapy to further decrease the synkinesis rate needs to be evaluated in larger clinical trials.

In case of extratemporal facial nerve reconstruction, for instance, after tumor resection and resection of tumor-infiltrated parts of the facial nerve, one option might be the reconstruction of the upper division of peripheral facial branches with the original facial nerve or with the masseteric nerve and of the lower division with the hypoglossal nerve using the jump technique. Even all three nerves and/or branches of the facial nerve from the contralateral side can be used (55–57). Using such a combined approach separates the upper and lower face by using different motor sources. As a result, synkinesis between the various motor supply areas is impossible.

There are a few pioneering publications performing facial nerve reconstruction combined with selective neurectomy (a technique originally applied after the occurrence of facial synkinesis, refer below). The idea is to target the regeneration of the nerve fibers to selected facial muscles to reduce the possibility of collateral sprouting in the direction of different facial muscles (58). The series is so far small, hence, the efficacy of this approach to reducing facial synkinesis should be further validated in future studies.

*Recommendation:* If a complex reconstruction of the facial nerve in the facial plexus region is needed, a combined approach that allows for a separation of the upper and lower face innervation to two or more motor nerves may be beneficial by preventing synkinesis between the upper and lower face.

### Therapy of facial synkinesis

The major therapy option for synkinesis is facial training, medical treatment, and surgery (5). Most publications of the last decade on therapy are dealing with botulinum toxin treatment. As it is a drug treatment, clinical trials with advanced methodology are much easier to design than facial training. Facial training (in the form of physiotherapy, neuromuscular retraining, cognitive therapy, and other techniques) has been used for a long time as a standard element for the treatment of both acute facial palsy and patients with synkinesis. This is important when interpreting studies on physical therapy, as the studies often do not clearly address at which stage of the disease the patients were treated (59). Furthermore, there are no international standard protocols for facial training. Studies of surgery for synkinesis are relatively scarce (to treat ocular synkinesis even scarcer), vary widely in surgical techniques described, suffer from small sample sizes, and include a wide variety of types of synkinesis (60). Second, surgery typically is not the first choice of treatment. Patients who no longer want to receive botulinum toxin injections every 4–6 months, typically ask for surgery. Secondary treatment failure due to the development of autoantibodies against botulinum toxin is a potential cause for failure yet has never been reported for a patient with facial synkinesis.

#### Facial training

**Figure 3:**
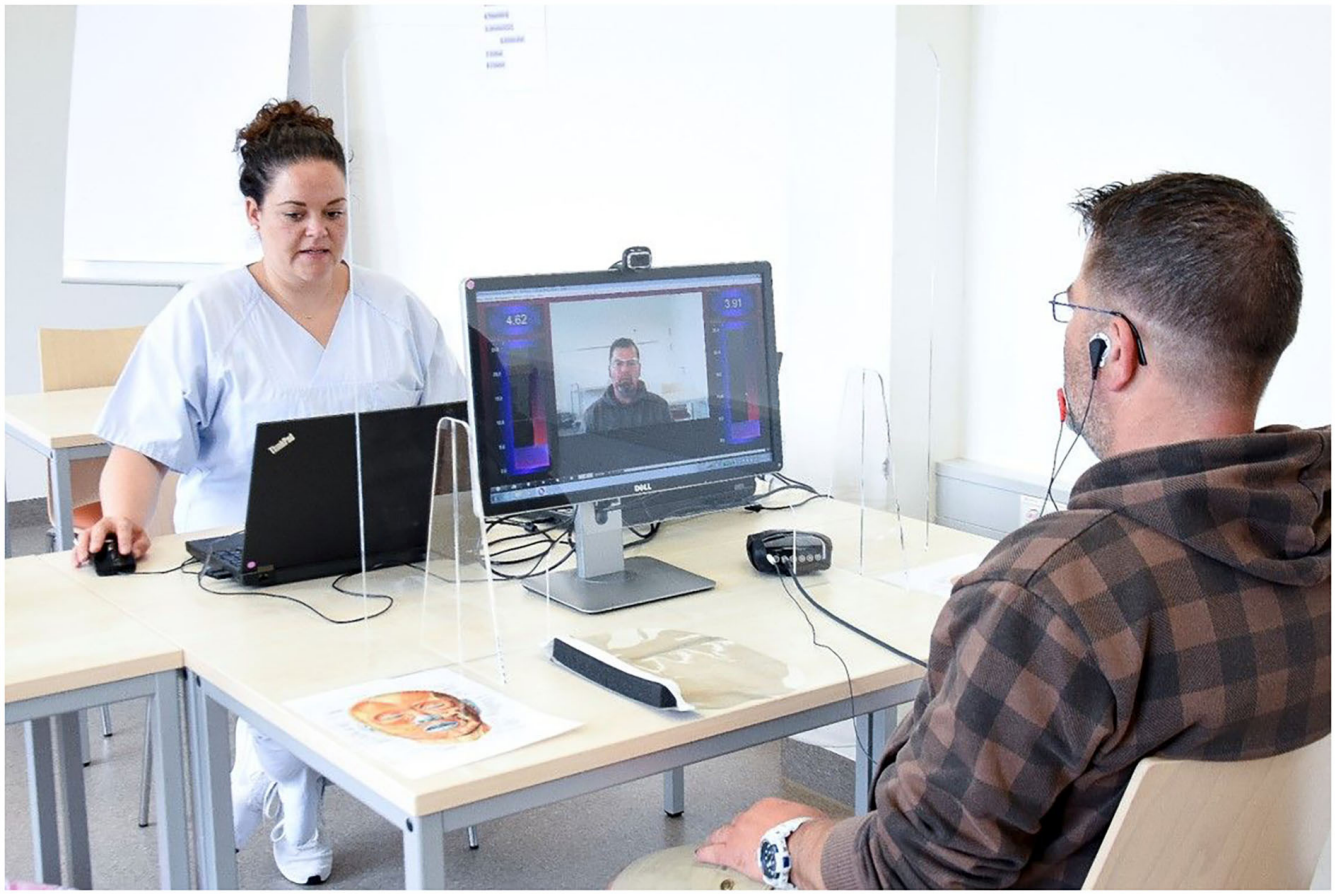
Optimal therapy setting for EMG feedback-based training. The patient is sitting in the front **(right side)**. Surface EMG electrodes are positioned on the training muscle (to be activated) and a synkinetic muscle (not to be activated). The patient sees himself on the computer screen, while the muscle activity is visualized in pseudo colors as activity bars. The therapist in the back **(left side)** sees the same information as the patient and is guiding the patient through the different mimic movement tasks.

It is important to differentiate facial training in the acute phase of facial palsy or for central facial palsy from physiotherapy for facial synkinesis. In the acute stage, physiotherapy uses passive techniques to reduce facial muscle tension and sensory stimulation to promote the reactivated motor skills. It includes active exercises as soon as the first movements are possible. In contrast, the aim of facial training is to reduce synkinetic movements with better control and increase intended movements (61). Table 4 shows an overview of the important elements of facial training for synkinesis. Facial muscles have a limited ability to provide feedback. Proprioceptive and joint receptors are absent in the face. Providing feedback is an essential element of modern physiotherapy for facial rehabilitation. A mirror, i.e., visual feedback, is deceptive support because it only shows the result of synkinesis, for instance, an incomplete asymmetric open smile, and suggests a persistent paresis of the muscles for lifting up the corner of the mouth. In fact, these muscles are contracted but counterbalanced by unintentional movements of antagonistic muscles. One possibility is to use surface EMG to show the patient the individual muscle activity during training (Figure 3). This EMG feedback is used to steer the training. EMG electrodes are placed on the muscle whose control ought to be improved (for instance, zygomatic muscle) and on the muscle which should not be moved synkinetically (for instance, orbicularis oculi muscle). The strength of EMG activity is visualized on a computer screen or by auditory feedback. The task for the patient is then to increase the activity of the intended muscle movement while keeping the activity of the synkinetic muscle to a minimum (61). Training sessions for one task typically range from 45 to 60 min. The idea is that the patient learns new corrected motor programs through intense repetitions. The contralateral side can also be included in the training as many patients try to compensate for malfunctioning movements by moving on the contralateral side. Here, the control electrode for minimal activity is placed on the contralateral target muscle. Once better facial movement control has been learned, home exercises without EMG feedback can be added. Facial EMG feedback training can effectively reduce synkinetic activity (61–63). Thus, EMG feedback can only be offered at the therapist's office. Home training systems have not yet been systematically evaluated and are not widely available in many countries (64).

**Table 4:** Typical elements of facial training against facial synkinesis based on this review.

**Element**	**Comment**
EMG feedback exercises	Use of surface EMG for visual and/or auditory feedback of muscle activity simultaneously recorded from facial muscles/movement to be trained and facial muscles whose synkinetic activity should be reduced. **Aim**: Improvement of facial movement control while reducing synkinetic movements.
Mirror exercises	In front of a mirror: soft specific movement (for instance, showing teeth), holding the position for 5 sec, then relaxing; and trying not to close the eye. **Aim**: Improvement of facial movement control
Other active exercises	Soft specific movement (for instance, closing the eye), holding the position for 5 sec, then relaxing; Hand on synkinetic area (for instance, corner of the mouth) **Aim**: Improvement of facial movement control.
Mime therapy	Combination of physiotherapy and mime training **Aim**: Improving symmetry of the face both at rest and during movement, simultaneously controlling synkinesis.
Passive exercises, stretching and massage	Stretching individual muscles, holding 10–30 s **Aim**: Reducing muscle tension
Tactile or vibratory stimulation	Hands or devices are used for sensory stimulation of the facial skin **Aim**: Hypothesis: Sensory perception and sensory stimulation should promote motor skills. More studies are needed to confirm such an effect.
Combination with botulinum toxin injections	Botulinum toxin injection into facial muscle whose synkinetic activity should be reduced before starting the facial training **Aim**: Making the exercises easier for the patient; improvement of facial movement control
**Other issues**	
Training session length	45–60 min, but also intensive training for 180 min established
Overall duration of the training	Typically over months, 10-day intensive training also established. Optimal duration not yet well studied.
Home exercises	If the patient shows improvement of facial movement control, home exercises can consolidate the therapeutic effect. 3–5 exercises twice daily. The additional effect of home training has still not been very well studied.
Telemedicine	Substitution of on-site training with therapist. The equivalence of telemedicine training has still not been very well studied.

Mime therapy is a combination of physiotherapy with mime training mainly offered in the Netherlands (65). Both components address not only functional movements but also emotional expressions mainly to promote symmetry of the face at rest and during movement, but also to control synkinesis. As with other forms of facial training, it also includes home exercises, massage, and relaxation exercises. The positive effect of mime therapy has been systematically evaluated (66, 67). Nevertheless, mime therapy has not been validated for facial synkinesis treatment outside the Netherlands.

*Recommendation:* Facial training is the basis of synkinesis therapy. The patient has to understand the underlying mechanisms for effective training, which differ from acute palsy therapy. The feedback that demonstrates the underlying muscle activity of muscles intended to move during the exercise, as well as the muscles with unintended synkinetic activity is the most effective component of facial training. EMG feedback is the most reliable feedback system and should be used if available.

#### Botulinum toxin injections

**Figure 4:**
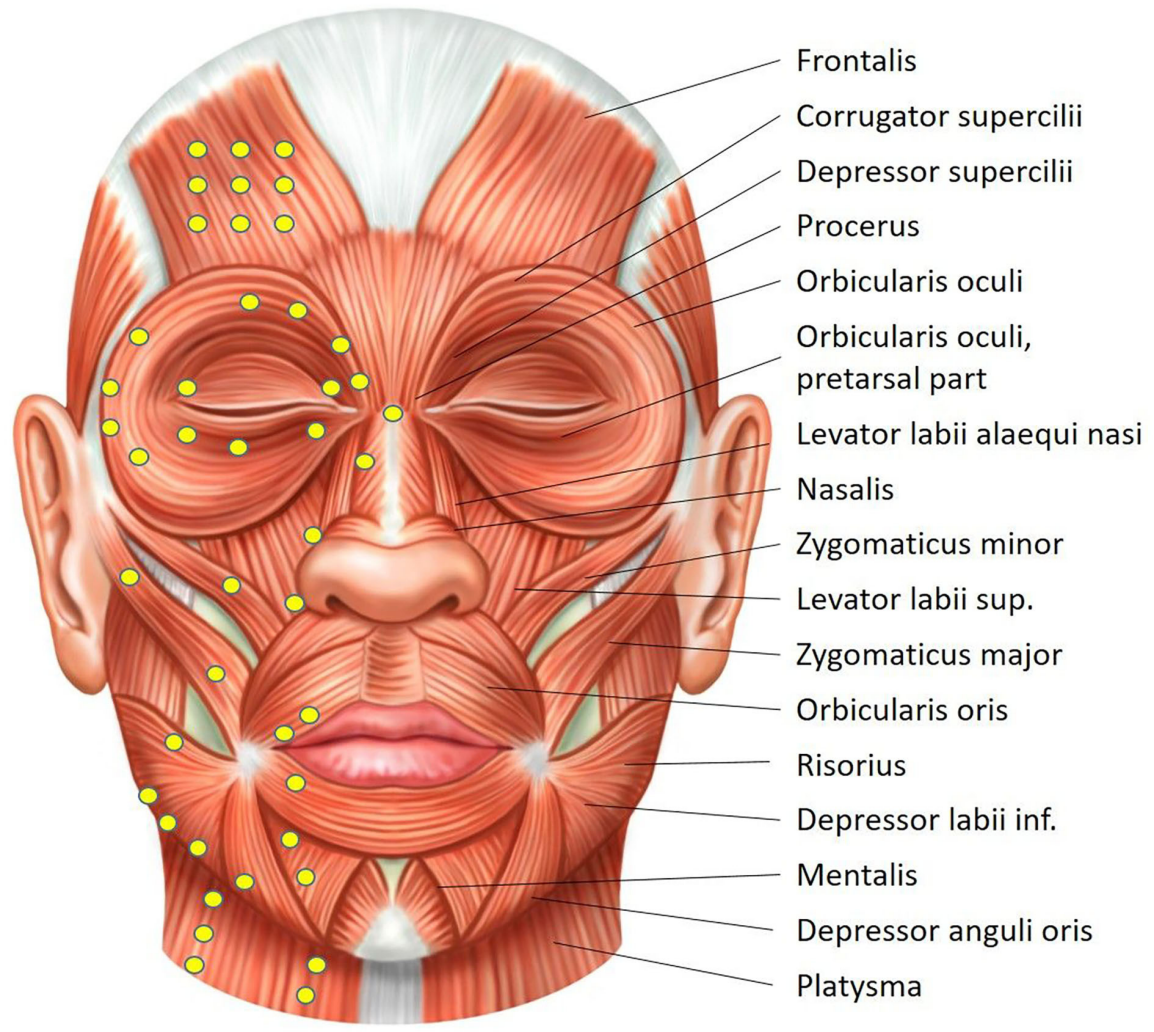
Overview of the facial muscles. Typical injection sites are displayed on the right hemiface. Drawing by Sonja Burger.

Botulinum toxin injections are an established and effective but off-label use therapy for patients with facial synkinesis worldwide (7, 68). The aim is to weaken a specific muscle movement that is synkinetically and involuntarily activated while a voluntary movement has to be preserved. Injections into another muscle counteracting a voluntary muscle movement can also increase the excursion of the intended movement (for instance, increased excursion of the ipsilateral smile following the treatment of the antagonistic acting of the ipsilateral depressor anguli oris muscle and of the platysma muscle). Another aim can be the weakening of a hyperkinetic muscle. All facial muscles can be targeted for botulinum toxin injections (Table 5). Most experience is published for the frontalis, corrugator supercilii, orbicularis oculi, levator labii superioris, zygomaticus major, orbicularis oris, risorius, buccinator, depressor anguli oris, depressor labii inferioris, mentalis, and platysma (Figure 4) (69). Even a transoral injection into the buccinator muscle can be helpful in patients with cheek biting, difficulty eating, and speech abnormalities (70, 71). An EMG guidance of the injections is not routinely performed but might be helpful if the initial treatment did not result in the desired effect.

**Table 5:** Typical target muscles for botulinum toxin injections and the effect of the treatment.

**Target muscle**	**Aim**
**Ipsilateral**	
orbicularis oculi, mentalis and platysma	Standard scheme for treatment of oculo-oral synkinesis
smile antagonists: depressor anguli oris, mentalis and platysma	Improve open smile
procerus, corrugator supercilii and frontalis	In addition, in cases with severe periocular synkinesis
buccinator	For midface hyperkinesis, can improve mastication, ease of emotional facial expression, cheek biting, and pronunciation of fricative consonants such as “s” and “f”
Paranasal muscles: levator labii superioris alaeque nasi, nasalis muscle, depressor septi, and depressor alae nasi	Improve open smile
**Contralateral**	
procerus, corrugator, mentalis, and depressor anguli oris	To optimize facial symmetry

Any other muscle affected by synkinesis beyond these very frequent target muscles can be the target of botulinum toxin therapy.

It is important to emphasize that synkinesis treatment is performed on the affected side. To achieve facial symmetry, injections of contralateral muscles (most frequent: procerus and/or corrugator, mentalis, and depressor anguli oris muscles) are also carried out (72). The contralateral treatment is not a synkinesis treatment. The injections begin with the treatment of a group of core facial muscles (Table 6) at lower doses and progressively increase until a steady state is achieved (73). In parallel, other muscles can be additionally treated or contralateral injections added (74, 75).

**Table 6:** Hints and typical dosages for botulinum toxin type A application for facial synkinesis.

**Topic/Muscle**	**Comment**
Contraindication	•History of hypersensitivity to any botulinum toxin constituent •Pregnancy •Breastfeeding •Neuromuscular junction disorders (myasthenia gravis), peripheral motor neuropathies •Active infections
Material	•Botulinum toxin type A •0.9% saline •30 G 4–10 mm needle (standard) •25 G 16 mm needle (platysma) •If necessary: lidocaine/prilocaine cream
Brand	•AbobotulinumtoxinA (ABO), vial with 500 IU •OnabotulinumtoxinA (ONA), vial with 100 IU •IncobotulinumtoxinA (INCO), vial with 100 IU
	Examples for reconstitution and concentration: •AbobotulinumtoxinA, with 2.5 ml 0.9% saline = 20 IU/0.1 ml •OnabotulinumtoxinA, with 2.0 ml 0.9% saline = 5 IU/0.1 ml •OnabotulinumtoxinA, with 2.5 ml 0.9% saline = 4 IU/0.1 ml •OnabotulinumtoxinA, with 4.0 ml 0.9% saline = 2.5 IU/0.1 ml •IncobotulinumtoxinA, with 2.0 ml 0.9% saline = 5 IU/0.1 ml •IncobotulinumtoxinA, with 2.5 ml 0.9% saline = 4 IU/0.1 ml •IncobotulinumtoxinA, with 4.0 ml 0.9% saline = 2.5 IU/0.1 ml **Important:** No exact dose conversion between abobotulinumtoxinA and onabotulinumtoxinA/ incobotulinumtoxinA is available. The dosing equivalency between is about 3–5 IU abobotulinumtoxin: 1 IU onabotulinumtoxinA/incobotulinumtoxinA
Injection	Subcutaneous, injection angle 45°
	Starting doses; recalculation to ABO; see above
Frontalis	ONA/INCO, concentration 4 IU/0.1 ml, 3–9 sites; total 6–18 IU
Corrugator supercilii	ONA/INCO, concentration 4 IU/0.1 ml, 1–2 sites; total 2–4 IU
Procerus	ONA/INCO, concentration 4 IU/0.1 ml, 1-2 sites; total 2–4 IU
Orbicularis oculi	ONA/INCO, concentration 4 IU/0.1 ml, 4 sites; total 5–8 IU
Zygomaticus	ONA/INCO, concentration 4 IU/0.1 ml, 1–2 sites; total 2–4 IU
Corner of the mouth	ONA/INCO, concentration 4 IU/0.1 ml, 3 sites; total 2–4 IU
Depressor anguli oris	ONA/INCO, concentration 4 IU/0.1 ml, 1–2 sites; total 1–2 IU
Mentalis	ONA/INCO, concentration 4 IU/0.1 ml, 1–2 sites; total 1–2 IU
Levator labii superioris	ONA/INCO, concentration 4 IU/0.1 ml, 1–2 sites; total 1–6 IU
Platysma	ONA/INCO, concentration 4 IU/0.1 ml, 3 sites; total 3–6 IU
Platysma, alternative scheme	ONA/INCO, concentration 2.5 IU/0.1 ml; 2.5 IU per injection site, 2-7 sites
Paranasal muscles: levator labii superioris alaeque nasi, nasalis muscle, depressor septi, and depressor alae nasi	ONA/INCO, concentration 5 IU/0.1 ml; 0.5–1.25 IU per injection site, 4 sites
Lacrimal gland	ONA/INCO, concentration 2.5 IU/0.1 ml; 2.5 IU per injection site, 1 site
Follow-up injections	Gradual adjustment of the botulinum toxin dose and the number of injection sites according to the therapeutic response in the prior sessions.

There are three brands of botulinum toxin type A products that are commercially available in Europe and the United States, and all are used for facial synkinesis treatment (72, 76): onabotulinumtoxinA (Botox, Allergan), abobotulinumtoxinA (Dysport, Ipsen, and Medicis), and incobotulinumtoxinA (Xeomin, Merz) (refer to Table 6 for dosages). Whether the selection of the brand has an effect on treatment intervals and dosage over time is not entirely clear (76). We could not identify a publication reporting on the use of botulinum toxin type B for synkinesis treatment (rimabotulinumtoxinB; NeuroBloc/MyoBloc, Sloan, or Supernus). The duration of effect is on average 3–4 months. Whereas the duration of the botulinum toxin effect seems to be stable over years, the number of injected muscles and the overall dosage needed seems to increase over time (72). There are no specific side effects of botulinum toxin used for facial synkinesis. There side effects are not different from those of other applications in the face. Side effects can occur when the dosage used is too high (leading to muscle paralysis) or when the drug is unintentionally infiltrating a neighboring muscle (leading to unintended muscle weakening or paralysis).

Botulinum toxin therapy can also be applied in combination with facial neuromuscular retraining, which has been shown to achieve long-lasting improvement (77, 78). The rationale behind it is that the botulinum toxin effect allows the patient to access the primary mimic musculature trained by physiotherapy with greater accuracy, improving practice patterns, and motor learning (52).

Botulinum toxin injection is also the (off-label) therapy of choice for gustatory hyperlacrimation (crocodile tears). Botulinum toxin is directly injected into the affected lacrimal gland. Optimal therapy leads to symptom-free periods of at least 6 months.

*Recommendation:* Botulinum toxin injections are a well-established treatment of facial synkinesis. One should start with a simple standard injection scheme to address ocular-oral synkinesis, using a low-dose regime to avoid side effects, which can increase if the desired effect is not reached. The treatment intervals should not be less than 4 months. One should always use the same brand with the same patient. Keeping records of dosage, injection sites, and therapy effects during follow-up allows us to adapt the injection strategy for optimal results. If needed, step-by-step, additional muscles can be included.

#### Surgery

Surgery is conventionally not regarded as the first-line treatment. Physical therapy and/or botulinum toxin treatment should be offered primarily. Typically, surgery is offered to patients wanting a definitive solution and no longer willing to receive botulinum toxin injections every 4–6 months. Surgery, which is irreversible by definition, should not be performed before synkinesis has reached its final state, usually 12–15 months after the onset of the lesion (46, 79), ideally, after 18 months, in which a final state is definitively reached. There are two different surgical procedures that can be performed to reduce facial synkinesis alone or in combination: selective facial muscle myectomy/myotomy and facial nerve branch neurectomy (Table 7).

**Table 7:** Typical targets for a facial muscle myectomy and facial nerve branch neurectomy.

Myectomy^*^	**Comment**
**Ipsilateral**	
Orbicularis oculi	2 incisions, upper incision along the upper lid crease if present, and lower incision along and 2 mm below the lower lid margin, to expose the orbicularis oculi muscle. Segmental resection of the pretarsus, and preseptum, but NOT of preorbital portion in the upper and lower eyelids
Depressor anguli oris	Intraoral resection incision *via* vertical mucosa on the inner side of the lower lip to dissect and resect the muscle
Platysma	2 parallel incisions, submandibular and above clavicle, to resect the platysma in the region in-between
Corrugator supercilii	Suprabrow incision for near-total resection
**Neurectomy**	
Improving smiling	Prerequisite: active mobility of zygomatic major and/or minor and levator muscles and facial nerve monitoring Classical preauricular incision to elevate a flap along the parotidomasseteric fascia to the anterior parotid border; alternatively a retrotragal rhytidectomy incision is used, followed by blunt dissection of a plane deep to the SMAS and platysma to access the distal branches of the facial nerve. Confirmation of undesired and desired movement via nerve stimulation. Identified branches are ligated and resected; can be combined with platysma myectomy
Improving brow function	Identify branches of the temporal branch of the facial nerve that innervate the corrugators and the superior orbicularis oculi with nerve stimulation about 1 cm lateral to the lateral canthus in the direction of superolateral orbital rim. Stimulation demonstrates a depression and medialization of the eyebrow. The main branches innervating the frontalis muscle must be preserved
**Muscle transfer**	
Depressor anguli oris transfer to depressor labii inferioris	After detachment of the depressor anguli oris muscle (see above), the muscle is transferred and attached to the weak depressor labii inferioris muscle

* The result of a myotomy instead of myectomy seems to be less predictable.

If muscle surgery is performed, most surgeons resect part of the target muscle (myectomy) (80–83). Although mentioned as a technique, myotomy is seldom reported, probably due to its limited predictability. In order to simulate the effect of myectomy, the target muscles can be blocked with local anesthesia. A local anesthesia block of the hypertonic depressor anguli oris muscle can demonstrate its inhibitory mimetic role in synkinesis. A depressor anguli oris muscle block can show and simulate an improved resting symmetry, a better modiolus angle, and exposure of teeth during a smile (80–82). The method can also be used to demonstrate to the patients the effect of a proposed myotomy.

The aim of neurectomy for more symmetrical smiling is to improve superolateral oral commissure excursion by the ablation of nerve branches that cause dysfunctional facial movements while preserving nerve branches that promote a natural smile (84). The procedure can also be combined with myotomy of the platysma. It is advantageous to perform the neurectomy in awakened patients after the dissection of the facial branches under general anesthesia (85). This allows better control over the weakening effect and reduces the risk of too much weakening. Synkinetic brow function with brow depression is the result of co-contraction of the frontalis muscle with the corrugator, procerus, and superior portion of the orbicularis oculi muscles (86). Neurectomy of the corrugators and the superior orbicularis oculi might improve the situation (86). Periocular neurectomy can produce a significantly wider palpebral fissure while smiling (60). Unfortunately, neurectomy cannot fulfill the aim to obtain a permanent solution: most patients, at least after periocular neurectomy, need to re-start treatment with botulinum toxin after a median time of about 1 year (60). There are not much long-term data after neurectomy is published.

If the patient has not yet received an upper eyelid weight during the phase of acute facial palsy, a re-evaluation is recommended. Periocular synkinesis, especially hyperkinesis, can erroneously pretend a sufficient upper lid function. Lid loading can improve malfunctioning blinking and thereby reduce the synkinesis effect (87). In the case of a hyperkinetic depressor anguli oris muscle and a weak depressor labii inferioris muscle, a transfer of the detached depressor anguli oris muscle (instead of myectomy only) has been proposed. However, the additional effect to improve smiling is not convincing (88).

As described above, reconstruction of the peripheral facial nerve with different motor nerves can prevent facial synkinesis. Furthermore, the first small series of studies are published using this approach *post-hoc*, i.e., when synkinesis has developed and if patients show severe eye closure during smiling. Due to these preliminary data, if these, an option is to cut the zygomatic branch (after intraoperative verification of zygomatic muscle contractions by nerve stimulation) and to reconstruct the motor supply of the zygomatic muscle by a masseteric-zygomatic nerve transfer (89), or by cross-facial nerve grafting (90).

**Recommendation**: Surgery in form of selective myectomy or neurectomy is a third-line treatment option and the decision should be individualized and done with caution, after discussing possible complications and limited success rate. Studies on the long-term effects are lacking.

**Figure 5:**
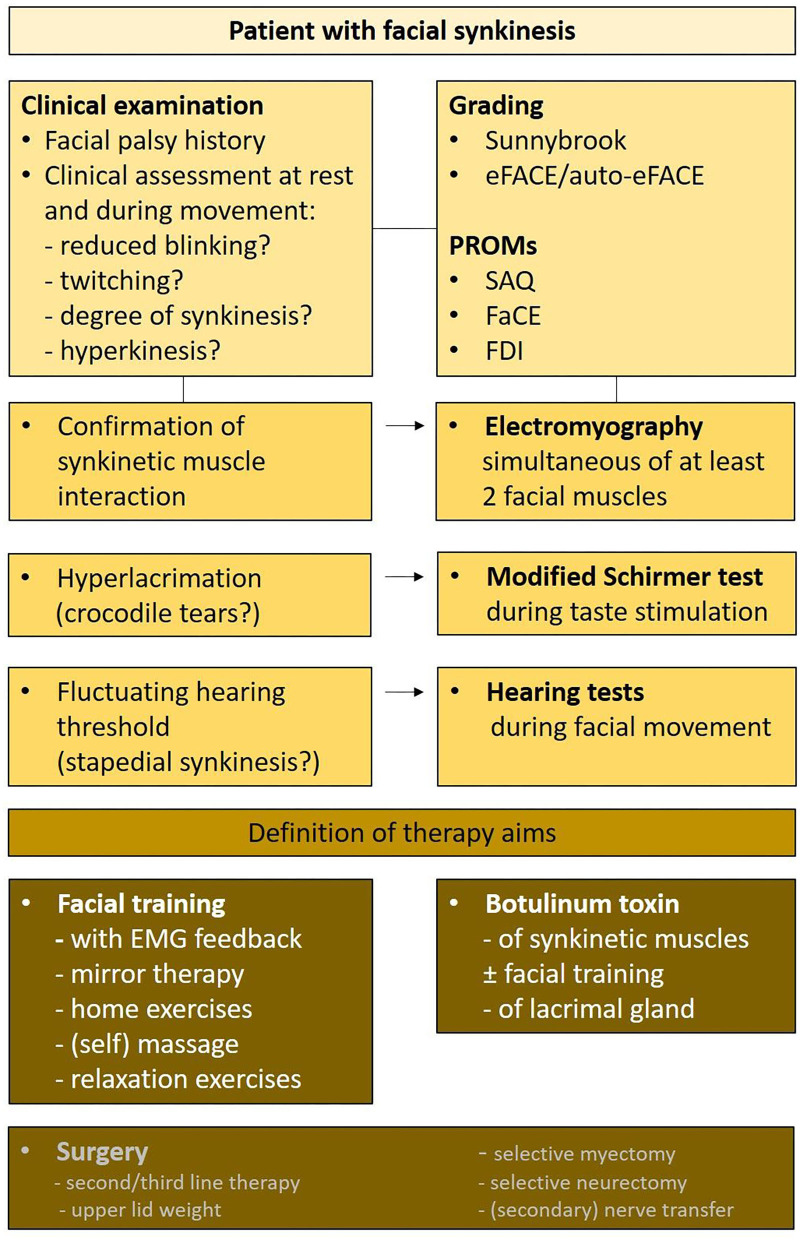
Therapy algorithm of all important diagnostics to define personalized therapy aims allowing to define the therapy strategy. PROM, patient-reported outcome measures; SAQ, Synkinesis Assessment Questionnaire; FaCE, Facial Clinimetric Evaluation; FDI, Facial Disability Index.

The entire workflow from diagnostics to therapy decisions based on the review and the authors' consensus is presented in Figure 5.

## Discussion

Improving the coordinated movement of facial muscles and better facial symmetry are the main aims of the treatment of facial synkinesis. Given the limited number of prospective studies, the heterogeneity in the treatment protocols, and the missing standardization of outcome assessments, it was not possible to carry out a meta-analysis to obtain a quantitative summary of the results. Furthermore, this systematic review showed that long-term data on treatment outcomes and data on the long-term quality of life of these patients are missing.

We have found some other limitations that may present challenges for future research: Facial synkinesis occurs in 10–20% of children after acute facial palsy (91). Principally, the same therapy strategies are offered to children (92). Facial training needs adaptation to the child setting to improve compliance. Outcome studies are sparse. More clinical studies are needed to construct more specific recommendations for the treatment of facial synkinesis in children.

Traditional facial training is linked to the traditional setting of a 45–60 min therapy session with the therapist. More intensive longer sessions over a shorter time seem to result in the same effect as traditional short sessions applied over months (63). Due to the limited number of specifically trained therapists, it is unlikely that we can realize a broader application of long therapy sessions in clinical routines. Nevertheless, the patients are generally highly motivated and have a continued interest in rehabilitation therapy. Furthermore, patients with facial synkinesis show a high interest in innovative digital solutions for facial rehabilitation (44). Boosted by the pandemic, the future focus may be on home-based sensor-based digital technology solutions with remote monitoring by the facial therapist, thus facilitating frequent intensive training sessions (93).

Moreover, an international standard is needed for comparable outcome measures to evaluate any new therapy concept for facial synkinesis. Thereby, it is not enough to apply the PROMs described in the diagnostics section of this guideline. What is needed is a consensus on minimal important changes (MICs) for facial synkinesis therapy (45).

The patients will profit—as in many other fields of botulinum toxin therapy—from newer formulations with longer duration of effect. It remains to be seen if, for instance, daxibotulinumtoxinA can fulfill these expectations (94). Finally, for any kind of surgical intervention, it is very important to pre-operatively define if a specific facial muscle that is disturbed by synkinesis has distally reachable nerve fibers that would allow a specific stimulation for a muscle contraction without synkinetic interference. Recently, it has been shown that mapping with transcutaneous electric stimulation allows locating of such nerve fibers in patients with synkinesis (95). It remains to be shown that such information can be used for a secondary nerve transfer to strengthen muscle activity *via* these well-defined peripheral nerve fibers. Furthermore, it is conceivable that implantable bionic devices are used in the future to improve specific mimic muscle functions *via* peripheral nerve stimulation in patients with synkinesis. More specific interventions addressing cortical re-organization in patients with synkinesis would also expend the therapy options.

## Conclusion

Facial synkinesis is a post-paralytic syndrome occurring in about 30% of patients after acute peripheral facial nerve palsy. Patients with facial synkinesis suffer from both aesthetic and functional deficits leading to decreased quality of life and high motivation for rehabilitation. An up-to-date guideline with recommendations for the optimal treatment of the patients is herewith published. The optimal approach includes a standardized assessment of the synkinetic symptoms including synkinesis-specific patient-reported outcome measures and electrophysiological definition of the individual synkinesis pattern. Optimal diagnostics lead to personalized treatment recommendations. First-line treatment is facial training including feedback elements (with or without EMG) followed by botulinum toxin injections according to standard regimes published here. Surgery is normally reserved for individual patients with unsatisfactory first/second-line treatment. Considering the low level of evidence and outcome, selection should be done with caution. Future diagnostic tests may include refined facial mapping, which may allow precise nerve transfers or electrostimulation by bionic devices to overcome synkinetic muscle function.

## Author's note

This article was written by members and invitees of the International Head and Neck Scientific Group (www.IHNSG.com).

## Data availability statement

The original contributions presented in the study are included in the article/supplementary material, further inquiries can be directed to the corresponding author/s.

## Ethics statement

Written informed consent was obtained from the individual(s) for the publication of any identifiable images or data included in this article.

## Author contributions

OG-L: conceptualization and literature review. OG-L and JP: literature analysis and first draft preparation. OG-L, JP, OC, AM, AS, OR, VV, and AF: Delphi rounds to find a consensus on the recommendations and writing, review, and editing. AF: supervision. All authors approved the final version.

## Conflict of interest

The authors declare that the research was conducted in the absence of any commercial or financial relationships that could be construed as a potential conflict of interest.

## Publisher's note

All claims expressed in this article are solely those of the authors and do not necessarily represent those of their affiliated organizations, or those of the publisher, the editors and the reviewers. Any product that may be evaluated in this article, or claim that may be made by its manufacturer, is not guaranteed or endorsed by the publisher.
